# Molecular Biology and Therapeutic Targets of Primitive Tracheal Tumors: Focus on Tumors Derived by Salivary Glands and Squamous Cell Carcinoma

**DOI:** 10.3390/ijms241411370

**Published:** 2023-07-12

**Authors:** Alessandro Marchioni, Roberto Tonelli, Anna Valeria Samarelli, Gaia Francesca Cappiello, Alessandro Andreani, Luca Tabbì, Francesco Livrieri, Annamaria Bosi, Ottavia Nori, Francesco Mattioli, Giulia Bruzzi, Daniele Marchioni, Enrico Clini

**Affiliations:** 1 Respiratory Diseases Unit, Department of Medical and Surgical Sciences, University of Modena Reggio Emilia, University Hospital of Modena, 41121 Modena, Italy; annavaleria.samarelli@unimore.it (A.V.S.); gaia.cappiello@gmail.com (G.F.C.); alessandreani@yahoo.it (A.A.); lucatabbi@gmail.com (L.T.); francescolivrieri@gmail.com (F.L.); annamaria.bosi94@gmail.com (A.B.); ottavia.nori.on@gmail.com (O.N.); giulibru92@gmail.com (G.B.); enrico.clini@unimore.it (E.C.); 2 Clinical and Experimental Medicine PhD Program, University of Modena Reggio Emilia, 41121 Modena, Italy; 3 Otolaryngology Unit, University Hospital of Modena, 41121 Modena, Italy; franz318@hotmail.com (F.M.); daniele.marchioni@unimore.it (D.M.)

**Keywords:** primary tracheal tumor, rigid bronchoscopy, radiotherapy, adenoid cystic carcinoma, treatment outcome, survival

## Abstract

Primary tracheal tumors are rare, constituting approximately 0.1–0.4% of malignant diseases. Squamous cell carcinoma (SCC) and adenoid cystic carcinoma (ACC) account for about two-thirds of these tumors. Despite most primary tracheal cancers being eligible for surgery and/or radiotherapy, unresectable, recurrent and metastatic tumors may require systemic treatments. Unfortunately, the poor response to available chemotherapy as well as the lack of other real therapeutic alternatives affects the quality of life and outcome of patients suffering from more advanced disease. In this condition, target therapy against driver mutations could constitute an alternative to chemotherapy, and may help in disease control. The past two decades have seen extraordinary progress in developing novel target treatment options, shifting the treatment paradigm for several cancers such as lung cancer. The improvement of knowledge regarding the genetic and biological alterations, of major primary tracheal tumors, has opened up new treatment perspectives, suggesting the possible role of biological targeted therapies for the treatment of these rare tumors. The purpose of this review is to outline the state of knowledge regarding the molecular biology, and the preliminary data on target treatments of the main primary tracheal tumors, focusing on salivary-gland-derived cancers and squamous cell carcinoma.

## 1. Background

Primary tracheal tumors are rare, constituting approximately 0.1–0.4% of malignant diseases; they are usually malignant (90%) in adults, while in children, they are rarer and are mostly benign (only 10–30% of malignant tumors) [1].

Squamous cell carcinoma (SCC) and adenoid cystic carcinoma (ACC) account for about two-thirds of all adult primary tracheal tumors, although a heterogeneous group of benign and malignant tumors may arise from the structures of the tracheal wall [2]. Diagnosis is often late because symptoms appear only when the central airway tumor has occupied more than 60% of the tracheal lumen [3]. Furthermore, symptoms are often mistaken for those of other respiratory diseases such as asthma or obstructive pulmonary disease (COPD), resulting in diagnosis delay and limiting the possibility of surgery due to the advanced stage of the disease. Surgical resection supplemented by postoperative radiotherapy is the treatment path of choice to achieve long-term survival and relieve airway obstruction [4]. Patients not suitable for surgery are managed with definitive radiation therapy. Local recurrence and extrathoracic metastasis are not uncommon and may require systemic treatment such as chemotherapy or target therapy [5]. Studies focused on the biological alteration of these tumors are limited. However, understanding the molecular alterations behind these tumors could improve the therapeutic management of non-resectable cancers by opening up the possibility of target treatments. The purpose of this review is to outline the state of knowledge regarding the molecular biology, and the preliminary data on target treatments of these rare tumors, focusing on salivary-gland-derived cancers and squamous cell carcinoma.

## 2. Primitive Tracheal Tumors Derived by Salivary Glands

### 2.1. Adenoid Cystic Carcinoma

#### 2.1.1. Pathological Features

Together with SCC, ACC represents the most frequent type or primary tracheal tumors. ACC arises presumably from the primitive tracheobronchial seromucinous glands, showing myoepithelial and ductal cell differentiation [5]. The first pathological and clinical description of primitive ACC of the trachea was drawn by Billroth in 1859 as a benign glandular neoplasm or adenoma [6]. However, ACC is now considered a low-grade malignant tumor due to its local invasiveness and ability to metastasize to distant organs. Histologically, ACC is a biphasic tumor comprised of ductal and myoepithelial components. Three histological growth patterns have been described [7]. *Cribriform* is the most common pattern, featured by uniform basaloid cells surrounding pseudocysts, arranged in well-defined islands of variable size, defining a “Swiss cheese” or sieve-like pattern. The cyst-like spaces are referred to as “pseudocysts” because they do not represent true glandular lumina and contain basophilic glycosaminoglycans. However, nests with true glandular lumina, composed of cells with ductal differentiation, are rarely observed in this growth pattern. The *trabecular pattern* is featured by eosinophilic hyalinized stroma surrounding basaloid cells arranged in thin strands, forming a tubular architecture. Well-formed ducts with epithelial and myoepithelial layers are more prominent than in the *cribriform pattern*. The *solid pattern* is featured by basaloid cells aggregated without the formation of tubular architecture or cysts. Furthermore, in this pattern, tumor cells appear larger, nuclear pleomorphism is more accentuated, and mitotic figures and comedonecrosis are usually seen [7]. ACC rarely presents a pure growth pattern, while more frequently the different patterns are mixed. Szanto P.A. et al. proposed a grading system based on the percentage of each pattern in tumor composition [8]. Grade I tumors contain only the trabecular or cribriform pattern without a solid component. Grade II tumors are composed of a trabecular or cribriform pattern associated with a solid growth pattern of less than 30%. Finally, grade III ACC is composed of a solid component of more than 30%. Although the results of studies are conflicting, the presence of a solid pattern is commonly considered a factor related to poor prognosis in patients suffering from ACC [9,10]. ACC is usually an indolent salivary gland carcinoma characterized by slow growth within the tracheal lumen; thus, diagnosis is often delayed for 5 or more years. However, ACC can occasionally undergo transformation to high-grade carcinomas, defining a pathologic condition named “dedifferentiated ACC” [11]. The term “dedifferentiated” is typically applied to sarcoma such as chondrosarcoma and liposarcoma showing a transition from a low-grade area to a more pleomorphic undifferentiated phenotype [12]. High-grade or “dedifferentiated” ACC contains two histologic patterns: an area of conventional ACC and an area of undifferentiated carcinoma or poorly differentiated adenocarcinoma. Histologic markers of this high-grade transformation in ACC are: the loss of biphasic ductal–myoepithelial differentiation, the presence of areas of necrosis, and a high mitotic rate [13]. Other findings include micropapillary and squamous growth patterns as well as fibrocellular desmoplasia. “Dedifferentiated ACC” is associated with an accelerated clinical course with a high propensity for lymph node metastases^13^. Despite the molecular mechanisms behind this transformation not being clear, p53 mutation seems to play a significant role [14].

#### 2.1.2. Molecular Biology of ACC

Seminal cytogenetic work by Sandros et al. reviewed a large sample of 189 benign and malignant salivary gland tumors, collected over 10 years [15]. A chromosome pattern featured by chromosome 6 deletion was observed in more than 50% of the ACC cases, suggesting a role in tumorigenesis through an oncogene activation or through a loss of tumor suppressor residing in this region [15]. Specifically, the breakpoints were clustered within the 6q22–25 region, causing a minimal common deletion of at least 6q25-qter. However, subsequent fluorescent in situ hybridization (FISH) studies found that the loss of 6q had been misinterpreted by banding studies. Indeed, the distal 6q “deletions” were a result of the translocations between the long arm of chromosome 6 and the short arm of chromosome 9. Persson M et al. showed a recurrent t(6;9)(q22–23;p23–24) translocation in ACC resulting in a fusion of the MYB oncogene to the transcription factor NFIB [16]. The MYB–NFIB fusion proteins retain the DNA-binding and transactivation domains of MYBand; it is therefore expected to activate MYB target genes. MYB belongs to a family of proteins that function as transcriptional regulators. MYB transcription factors are highly conserved from plants to vertebrates, suggesting a fundamental role in cellular homeostasis through different mechanisms such as the control of cell proliferation, apoptosis and cell differentiation. Furthermore, MYB plays a critical role in mammalian hematopoiesis, as indicated by its ability to regulate fetal hemoglobin expression and by embryonic lethality due to hepatic hematopoiesis in case of MYB disruption [17,18]. However, MYB activity is essential also for cell duplication through cyclin-dependent kinase (Cdks) expression and activity regulation [19]. MYB family members are often aberrantly expressed in human cancer, emphasizing their key role in the initiation and maintenance of cancer. Hyperexpression of MYB was first described in leukemic cells and more recently in solid cancers. However, the overexpression of wild-type MYB is not able to promote epithelial cell transformation, and genomic rearrangements is required [20]. In ACC, chromosomal rearrangements, i.e., t(6;9)(q23;p23), result in the translocation of strong enhancers near MYB or the MYBL1 locus, which activates their transcription. Translocation may also cause a loss of genetic material; therefore, it can be assumed that the consequent loss of tumor suppressor gene may play a role in ACC pathogenesis. However, an effort to identify a tumor suppressor gene at chromosome 6 loci, a typical site of deletions in ACC, was unsuccessful [21]. The molecular mechanisms by which MYB is activated by the t(6;9) translocation is still obscure. The gene fusion results in minimal MYB lost in exon 15 including the 3′-UTR, which contains several highly conserved target sites for miR-15a/16 and miR-150 micro-RNAs. These microRNAs were recently shown to regulate MYB expression negatively. Therefore, it was assumed that the deregulation of MYB may be the result of a loss of binding sites for negatively regulating miRNAs [16]. However, Drier et al. examined the genomic loci translocated to MYB in patients suffering from ACC and identified several super-enhancers in the rearranged portion of NFIB, which is able to interact with the MYB promoter, causing its overexpression [22]. This observation suggests that promoted–enhancer interactions may play a critical role in ACC tumorigenesis. Studies focused on fusion-positive tumors showed the overexpression of MYB target genes such as genes associated with cell cycle control (CCNB1, CDC2 and MAD1L1), apoptosis (AP15, BCL2, BIRC3, HSPA8 and SET), cell growth (MYC, KIT, VEGFA, FGF2, CD53), and cell adhesion (CD34) [16]. However, MYB is also highly overexpressed in ACC tumors without detectable chromosomal translocation. Frerich C.A et al. provided evidence that ACC tumors may use an alternative MYB promoter, named TSS2, leading to the expression of N-terminally truncated MYB proteins (ΔN Myb) with distinct functional activities [23]. The expression in cells of these Myb proteins with an N-terminal deletion changes the spectra of genes activated, leading to altered expression of hundreds of genes with a large impact on the activity of the oncoprotein [23]. RNA-seq analyses conducted by Freich C.A et al. showed that ΔN-Myb-expressing cells displayed activates SEMA4D signaling, which is implicated in invasiveness and perineural invasion and is correlated with ACC patient outcomes [23].

#### 2.1.3. Consequences of Myb Overactivation in ACC

**Figure 1:**
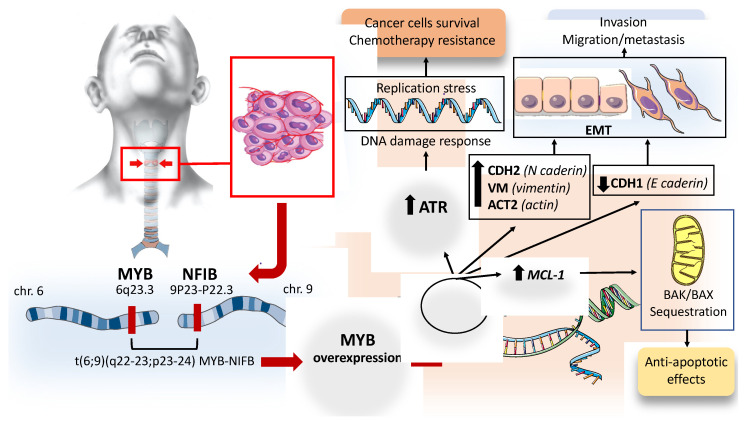
Molecular alterations behind ACC pathogenesis.

Activation of the master transcriptional regulator MYB through chromosomal translocation, copy number gain, or enhancer hijacking is the genomic hallmark of ACC. The consequences and downstream activation pathways due to the fusion protein or MYB overactivation in ACC cells have been analyzed in some studies. Andersson M.K et al. investigated the transforming potential and molecular consequences of MYB and MYB–NFIB overexpression in human mammary epithelial cells and cultured ACC cells [24]. This study showed that overexpression of MYB and MYB–NFIB fusion have analogous cellular consequences for cell proliferation and downstream target gene activation, both leading to the transformation of human glandular epithelial cells. Furthermore, they identified the DNA-damage sensor kinase ataxia telangiectasia and Rad3-related (ATR) as a MYB downstream target that is overexpressed in primary ACCs and ACC-patient-derived xenografts [24]. Genome instability is a hallmark of cancer, and DNA replication is the most vulnerable process that can lead to it. Normal cells are able to respond to DNA damage through different mechanisms of repair and through the activation of the checkpoint kinases. The coordination process of these complex mechanisms is known as the DNA damage response (DDR) [25]. Any condition leading to a high level of DNA damage will result in replication stress, a phenomenon characterized by DNA synthesis slow down and/or replication fork stalling. Replication stress is the primary cause of genome instability and is a typical feature of pre-cancerous and tumor cells. ATR is one of the main components of cellular DDR activated by replication stress. Oncogene expression drives cell proliferation by interfering with the controlling pathways of the cell cycle, resulting in replication stress and in a constitutive DDR activation, thus providing a link between replication stress and tumorigenesis [25]. Different observations showed that the loss of ATR pathway activity is lethal upon oncogene-induced replication stress or a lack of p53 function [26,27]. Upregulation of the ATR pathway has been described in several tumors and is required for cancer-cell survive under a replication stress environment [28,29]. Andersson M.K et al. showed that MYB and MYB–NFIB activate a significant number of DNA repair genes and genes involved in cell cycle control, promoting DDR [24]. This mechanism can partially explain the resistance to genotoxic stress typical of ACCs. However, coregulation of MYB and ATR is not restricted to ACC but has also been described in other human malignancies such as acute myeloid leukemia, adult T-cell acute lymphoblastic leukemia and colon carcinoma. All of these data suggest that the main function of ATR pathway overexpression in ACC is to promote tumor cell survival through an anti-apoptotic function at the level of mitochondria [30]. In support of this hypothesis, the treatment of cultured MYB-positive ACC cells with phase 2 ATR kinase inhibitor VX-970 results in significant tumor growth inhibition and apoptosis [24]. These data indicated that the inhibition of ATR pathway could be an interesting future therapeutic option in ACC with co-expression of MYB and ATR. The consequence of MYB overexpression in ACC cells was also explored by Xu et al., using the ACC cell line SACC-83 [31]. The authors overexpressed MYB, using a lentiviral vector, and knocked down MYB, using siRNA, in ACC cells, which showed that MYB overexpression promoted ACC cell proliferation, migration and invasion, whereas its knockdown inhibited these activities [31]. Furthermore, MYB overexpression was associated with the downregulation of CDH1 (the gene that encodes cadherin-1; E-cadherin) and upregulation of CDH2 (the gene that encodes cadherin-2; N-cadherin), VIM (the gene that encodes vimentin) and ACT2 (the gene that encodes actin, aortic smooth muscle), suggesting that MYB promotes epithelial–mesenchymal transition (EMT). EMT is a complex transformation process that induces local and distant progression of many cancers through a cellular differentiation from polarized epithelial phenotype to mesenchymal features; thus, the cells gain an invasive phenotype and stronger motility. The EMT process is marked by a loss of E-cadherin and by the expression of mesenchymal markers such as vimentin and N-cadherin [32]. Therefore, the results of Xu et al.’s study suggest that MYB regulated ACC metastasis by promoting EMT. Furthermore, the effect of MYB on tumor metastasis promotion was also investigated in an animal model, in vivo, through an injection of MYB overexpressing cells or control cells into the mouse tail vein [31]. The lung tissues were collected from all mice after 8 weeks. The mice injected with MYB-overexpressing cells had visible tumor nodules, which were not observed in mice treated with control cells; suggesting a key role of MYB in the promotion of lung metastasis [31]. MYB is able to decrease apoptosis and promote cancer cell survival through (*vi*) upregulation, which encodes proteins belonging to the BCL-2 family [33]. The BCL-2 proteins have conserved BCL-2 homology (BH) domains and are classified as pro- or anti-apoptotic proteins. The BCL-2-associated X protein (BAX) and the BCL-2 homologous antagonist killer (BAK) are pro-apoptotic multi-domain members that contain several conserved BH domains and act as apoptosis executors in mitochondria. MCL-1 is widely expressed in human cells and is located in the mitochondrial membrane via a hydrophobic tail [33]. MCL-1 exerts its anti-apoptotic function by sequestering BAK/BAX proteins [33]. Given the critical role of the BCL-2 family protein in maintaining cellular homeostasis, perturbation of these complex control mechanisms through the upregulation of anti-apoptotic MCL-1 protein can promote tumorigenesis. The overexpression of MCL-1 is described in several human cancers such as non-small cell lung cancer, breast cancer, ovarian cancer and pancreatic cancer [34]. MCL-1 upregulation may also play a significant role in cancer cell survival and drug resistance in ACC with MYB overexpression. Furthermore, in ACC cells, MYB is able to promote the migration and invasion of cancer cells through the upregulation of VEGFA and ICAM-1, and by increasing MMP7 and MMP9 expression [31]. Figure 1 summarizes the consequence of MYB activation in ACCs.

Translocations between the long arm of chromosome 6 and the short arm of chromosome 9 result in the fusion of the MYB oncogene to the transcription factor NFIB. The biological consequence of MYB–NFIB fusion is the overexpression of MYB with the activation of the target genes promoting cell survival, invasion and chemotherapy resistance. Read the text for details. ATR: ataxia telangiectasia and Rad3-related (ATR), CDH2: Cadherin 2, VIM: Vimentin, ACT2: Actin Alpha 2, Smooth Muscle, CDH1: Cadherin 1, MCL-1 myeloid cell leukemia-1, BAX: BCL-2-associated X protein.

#### 2.1.4. Receptor Tyrosine Kinase and Growth Factors Expression in ACC

Several tyrosine kinase receptors and growth factors are overexpressed in ACC. One of them is c-KIT, whose mutations affect signaling pathways such as NOTCH, PI3K Catalytic Subunit Alpha, and PTEN, as well as alterations in chromatin remodeling genes [35]. c-Kit is a receptor tyrosine kinase whose dysregulate function caused by either overexpression or mutation is involved in the development of several cancers such as hematological malignancies, thyroid cancer and breast cancer [36]. c-KIT, mapped to chromosome 4q11–12 in humans, was discovered in 1986 as the cellular homolog of the transforming viral oncogene v-kit in the Hardy–Zuckerman 4 feline sarcoma virus [37]. Binding of c-KIT to its ligand, stem cell factor (SCF), results in c-KIT homodimerization and autophosphorylation of selected tyrosine residues with activation of downstream signaling pathways. Signal cascade due to c-KIT activation is implicated in several physiological processes, including cell proliferation and survival. c-KIT downstream signaling results in the activation of mitogen activated protein kinase (MAPK)/extracellular signal-regulated kinase (ERK) pathway, which is involved in the regulation of gene transcription, promoting cell proliferation and exerting an anti-apoptotic function [38]. Cell survival, proliferation and evasion of apoptosis can also be promoted by the activation of phosphatidylinositol 3kinase/protein kinase B (PI3K/AKT) and phospholipase-C-γ (PLC-γ) pathways [38]. In addition, phosphorylation of some c-KIT tyrosine residues leads to the activation and translocation of STAT protein into the nucleus, promoting the transcription of target genes, which has an influence on growth, survival, apoptosis and differentiation functions [39]. c-KIT alterations resulting in “gain of function” such as mutation that led to constitutive activation of c-KIT in an SCF-independent manner represent an oncogenic driver in the development of several cancers including gastrointestinal stromal tumor (GIST), melanomas, mastocytosis and acute myeloid leukemia [38]. Vila et al. studied 14 adenoid cystic carcinoma of the salivary gland and performed mutational analyses of c-KIT by polymerase chain reaction, clonal selection and DNA sequencing [40]. c-KIT missense point mutations were detected in 88% of cases, including gain-of-function mutations in exon 11, and less frequently in exon 9, 13 and 17 [40]. However, Jeng Y-M et al. analyzed c-KIT expression and genetic alteration in the juxta-membrane domain (exon 11) and tyrosine kinase domain (exon 17) in 79 carcinomas of major and minor salivary glands without identifying gene mutation by DNA sequencing [41]. A previous study by Holst V A et al. noted Kit protein expression in 90% of ACCs with an association between the presence of at least 50% Kit-positive neoplastic cells and Grade 3 ACC or a solid growth pattern [42]. However, no c-KIT mutations were found in any of the tumors examined [42]. Freier K et al. conducted a study aimed at clarifying if the overexpression of c-KIT protein in ACC was due to an increase of gene copy number as in other tumors such as glioblastoma and small-cell lung cancer [43]. Tumor tissue microarray sections of 55 ACCs were analyzed by fluorescence in situ hybridization (FISH), which revealed copy number gains of the KIT gene in 6.1% of the tumors [43]. In conclusion, the reason for kit overexpression in ACC remained unclear, the point mutations presumably being rare events, while KIT copy number gains may contribute only to a limited subset of ACC. However, due to the high c-KIT expression in ACC, target therapies against c-KIT receptor using tyrosine kinase inhibitors have been undertaken in some clinical trials with negligible clinical benefit. Epidermal growth factor receptor (EGFR) is a transmembrane tyrosine kinase receptor that showed “gain of function” in many cancers through different mechanisms such as receptor overexpression, mutations, ligand-dependent receptor dimerization and ligand-independent activation [44]. EGFR signaling cascade is a key regulator in cell proliferation, differentiation, survival and cancer transformation [44]. EGFR inhibition is one of the main strategies of treatment in cancer with abnormal activation of this tyrosine kinase receptor such as non-small lung cancer [45]. Dahse R et al. analyzed 65 salivary gland carcinomas, among which 25 were ACCs. In ACC samples, the immunohistochemical expression of EGFR was quantified as weak–moderate in 32% and as strong in 64% of samples [46]. However, EGFR gene mutation is rarely found in ACCs [47]. The lesson learned from lung cancer is that the concurrent presence of EGFR and Kristen rat sarcoma viral oncogene homologue (KRAS) mutations is rare and their appearance is believed to be mutually exclusive [48]. Furthermore, tumors harboring KRAS mutation showed resistance to tyrosine kinase inhibitor therapy in different studies [48]. The KRAS gene is a member of the rat sarcoma viral oncogene family (RAS) and is the most common oncogenic gene driver in human cancer [49]. RAS proteins are GTPases located downstream of EGFR, which act as regulators of the EGFR pathways. Gene mutation, typically at codon 12, 13 or 61, may result in constitutive activation of RAS and is associated with poor prognosis in many cancers. Saida K et al. analyzed the mutational status of the EGFR pathway genes in 70 cases of ACCs, finding KRAS mutation in 14.3% of cases [47]. Furthermore, KRAS mutation was associated with poor prognosis for the patient [47]. Several studies have analyzed EGFR pathway inhibition treatments in patients suffering from ACC, using different therapeutic agents such as gefitinib, cetuximab, and lapatinib, without finding a meaningful response rate [50]. Vascular endothelial growth factor (VEGF) is an angiogenic factor upregulated in several cancers with a key role in tumor angiogenesis. Overexpression of VEGF is correlated with malignant disease progression and metastasis [51]. In ACC, high VEGF expression was correlated with the tumor stage and microvessel density [52]. In a study by Park S et al., 68 patients underwent curative surgery, and the available tissue samples were analyzed [53]. High expression of VEGF was correlated with poor prognostic factors for overall survival. The Notch signaling pathway is a regulator of self-renewal and differentiation in several tissues, and its dysregulation is implicated in oncogenic transformation [54]. The Notch family comprises four transmembrane protein receptors (Notch1 to 4) and five ligands, which are members of the Delta-like (DLL1 to 4) and the Jagged (JAG1 and JAG2) family. The interaction between the Notch receptor with Delta-like and jagged ligands on neighboring cells results in downstream signaling activation through the initiation of the sequential receptor proteolytic cleavages. ADAM10, a cell surface protein member of the ADAM family metalloprotease, cleaves the receptors on the cell membrane, releasing the Notch extracellular domain (NECD), while the transmembrane region of the Notch receptor is cleaved by γ-secretase, generating the Notch intracellular domain (NICD), which enters the cell nucleus and induces gene expression [55]. In the nucleus, target gene expression is promoted by the formation of a ternary complex consisting of NICD with the DNA-binding protein CBF1/RBPjk/Su (H)/Lag1 (CSL), which helps with adaptor protein Mastermind-like (MAML) transcriptional coactivator recruitment [55]. Despite point mutations that result in a gain of function in terms of Notch signaling possibly being relevant for oncogenic events in several hematologic and solid cancers, the true functional impact of Notch dysregulation in the context of cancer is not yet fully understood. For example, in glioma, several studies showed that Notch pathway dysregulation may act as both a tumor suppressor and oncogene [56]. However, in several solid cancers, such as colorectal carcinoma and pancreatic cancer, activating mutant Notch receptors may further collaborate with p53 mutations, promoting EMT, enhancing cancer invasiveness [57]. Furthermore, Notch mutations associated with gain of function are able to promote EMT, more aggressive cancer phenotypes, and gefitinib acquired-resistance in NSCLC [58]. NOTCH1 mutations with gain of function are present in approximately 20% of ACC cases, and are associated with more aggressive phenotypes, increased tendency to metastasize, and an overall shorter survival [59]. Ferrarotto R et al. genotyped 102 ACCs that have available pathologic and clinical data, showing that NOTCH1 mutations define a distinctive aggressive ACC subgroup associated with a significantly higher likelihood of solid subtype, advanced-stage disease at diagnosis, higher rate of liver and bone metastases, shorter disease-free survival and shorter overall survival when compared with NOTCH1 wild-type tumors [59]. Chintakuntlawar et al. retrospectively reviewed genetic testing for mutations by Foundation Medicine (Cambridge, MA, USA) performed on specimens derived from twenty-three patients suffering from ACC and identified 41 unique genes demonstrating either mutations or amplifications, among which 22% (5/23) were NOTCH mutations [60]. Su B et al. analyzed, in a SACC cell line, the effects of NOTCH1 overexpression or NOTCH1 knockdown on proliferation, migration and invasion. The knockdown of NOTCH1 in SACC cells significantly inhibited cell migration and invasion; in contrast, the overexpression of NICD1 promoted cell motility and invasiveness and robustly increased cell proliferation over the long term in vitro [61]. Furthermore, to further assess the oncogenic effect of NOTCH1 on tumorigenicity in vivo, Su B et al. inoculated subcutaneous SACC cells, knock-down for NOTCH1, silenced by NOTCH1-specific siRNAs, into the flanks of athymic mice, demonstrating that by silencing the NOTCH1 gene, tumor growth may be inhibited by inducing cellular apoptosis [61]. All of these data suggest that NOTCH1 gain of functions act as oncogenes in ACC pathogenesis and may be a potential treatment target.

**Figure 2:**
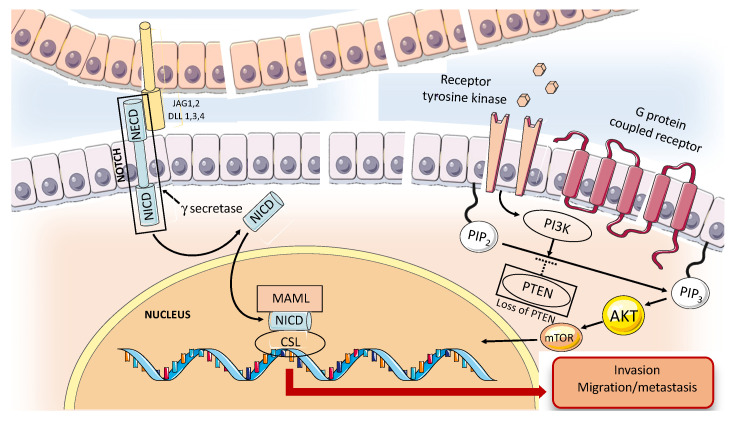
Molecular alterations associated with ACC aggressive phenotype.

Phosphatase and tensin homolog (PTEN) is a key tumor suppressor gene that acts by promoting cellular apoptosis and inhibiting cellular growth by antagonizing phosphatidylinositol 3-kinase (PI3K) signaling. Loss or alteration of PTEN function has been identified in several solid cancers and results in the activation of downstream components of PI3K pathway such as AKT and mTOR [62]. Liu et al. conducted an immunohistochemistry study on a total of 114 human salivary gland tumors, reporting that loss of PTEN expression is a frequent event in poorly differentiated, high-grade ACC (i.e., solid ACCs) [63]. Furthermore, reduced expression of PTEN in human SACC cell lines correlated with migration and invasion in vitro and in an animal model. The use of a PI3K/mTOR inhibitor named NVP-BEZ235 caused strong growth and invasion inhibition both in vitro and in a xenograft mouse model, suggesting a potential role of treatment in ACCs with loss of PTEN expression [63]. PI3K/PTEN/mTOR pathway could have a key role in ACC pathogenesis, as suggested by Ho et al., who determined the ACC mutational landscape identifying recurrent mutations in the FGF-IGF-PI3K pathway (30% of tumors) [64]. Figure 2 summarizes the major molecular mechanisms associated with the aggressive ACC phenotype.

NOTCH1 mutations with a gain of function are present in approximately 20% of ACC cases. The interaction between the Notch receptor with DLL and JAG on neighboring cells results in downstream signaling activation through the initiation of the sequential receptor proteolytic cleavages. The transmembrane region of the Notch receptor is cleaved by g-secretase, generating the Notch intracellular domain (NICD), which enters the cell nucleus and induces gene expression through the formation of a ternary complex consisting of NICD with CSL and MAML. Transcription of target genes ultimately results in the activation of the EMT process, with improvement in migratory and invasive cell properties. PTEN is a key tumor suppressor gene that acts by promoting cellular apoptosis and inhibiting cellular growth by antagonizing PI3K signaling. Loss or alteration of PTEN is a frequent event in high-grade ACC and results in the activation of the downstream components of the PI3K pathway, such as AKT and mTOR. NECD: Notch extracellular domain, NICD: Notch intracellular domain, DLL: delta-like ligands, JAG: Jagged ligands, MAML: adaptor protein Mastermind-like, CSL: DNA-binding protein CBF1/RBPjk/Su (H)/Lag1, PTEN: phosphatase and tensin homolog, PI3K: phosphatidylinositol 3-kinase, mTOR: mammalian target of rapamycin.

#### 2.1.5. Immune Checkpoint Targets in ACC

CD8^+^ tumor-infiltrating lymphocytes (TIL) are the key cells of the antitumor response through the release of enzymes such as perforin and granzyme B (Grb), which induce apoptosis in cancer cells. This anti-cancer immune response depends on the recognition and capture of tumor antigens by dendritic cells (DC), which have the task of transporting and presenting antigens to T lymphocytes in regional lymph nodes. Failure of this mechanism occurs due to the tumor’s ability to inhibit the immune activation of antigen-presenting cells, CD8^+^TIL and Natural Killer (NK), and involve some trans-membrane proteins such as Programmed Cell Death protein 1 (PD-1) and its ligand PD-L1. Cancer immunotherapy has been developed with the aim to promote an effective immune response against cancer cells through the inhibition of “cancer immune escape” mechanisms. PD-1 is a trans-membrane protein that plays a key role in inhibiting immune responses and promoting self-tolerance through the modulation of T-cell activity [65]. PD-1 is expressed on activated NK, B lymphocytes, macrophages, DCs, and monocytes and is highly expressed on tumor-specific T cells. PD-1 ligand (PD-L1 also referred to as CD279 and B7-H1) is a 33kDa type1 transmembrane glycoprotein usually expressed by macrophages, some activated T and B cells, DCs, and some epithelial cells under inflammatory stimuli. However, PD-L1 is overexpressed in tumor cells, acting as one of the main mechanisms for the promotion of the escape from the anti-tumor immune response, via binding to its receptors (PD-1) [65]. Indeed, the interactions between PDL-1 and PD-1 result in a reduction in the proliferation of PD-1-positive cells and their apoptosis. James P. Allison and Tasuku Honjo won the 2018 Nobel Prize for Physiology or Medicine for discovering a potential therapeutic treatment based on immune checkpoint inhibition in order to reactivate a T cell immune response against cancer cells [66]. Targeting PD-L1 has been associated with a significant clinical response in a wide range of solid cancers such as lung cancer, breast cancer and others [67]. Limited studies have investigated the expression and the role of the PD1/PD-L1 axis as an immune-escape mechanism in ACC. Mosconi et al. investigated the expression of the immune-related markers in 36 samples from patients with ACC as well as the associations between the immunological microenvironment characterization and the clinical features [68]. The main finding of the study is that the ACC microenvironment exhibits low immunogenicity, as evidenced by the low TIL and DC densities as well as low density of PD-1-positive cells and absence of PD-L1 cells [68]. Teng et al. proposed a classification based on immunological features of the cancer microenvironment, distinguishing four different cancer immune environments: type I: immune resistance, type II: immune ignorance, type III: intrinsic induction, and type IV: immune tolerance [69]. The majority of ACC cases reported by Mosconi et al. can be classified as type IV. In another study, Sridharan et al. examined tissue from 28 ACC samples obtained from 21 patients for immune cells and PD-L1/PD-L2 expression [70]. Most of the tumors showed few infiltrating immune cells, confirming the low immunogenicity of the ACC-related microenvironment, emphasizing that the lack of immune-cell infiltrate was associated with the expression of genes in the β-catenin/Wnt and PI3K pathways [70]. However, although no patients showed significant expression of PD-L1 on tumor cells, the authors found that PD-L2 was expressed in 60% of the primary and 73% of metastatic tumor samples. The role of PD-L2 expression in the modulation of the anti-cancer immune response is less explored than PD-L1. However, like PD-L1, PD-L2 is also able to bind PD-1 receptors, inhibiting T cell proliferation, and also binds to repulsive guidance molecule B (RGMb) receptor on the surface of several immune cells, promoting immune tolerance [71,72]. Tapias et al. conducted a retrospective review of specimens obtained by 23 patients with resected primary tracheal malignant tumors to determine the expression of PD-L1 and the infiltration of CD8^+^ immune cells in the tumor or peritumoral stroma [73]. PD-L1 expression was observed in 75% of SCC and 100% of adenosquamous carcinoma, but it was absent in ACC and mucoepidermoid carcinomas. Furthermore, the presence of CD8^+^ infiltration in the tumor stroma was significantly higher in cases of tracheal tumor with an SCC component than in salivary-type tumors [73].

#### 2.1.6. Current Treatment Strategy for ACC

The mainstay of ACC treatment is surgical resection. The indication for surgery treatment is determined based on local extension in the trachea and extension to contiguous organs, while the lymph node status does not influence survival and therefore does not contraindicate surgery [1]. Surgical resection may be also considered in metastatic disease, since the slow progression allows a long survival in patients presenting with distant metastases at the time of diagnosis. The surgical technique depends mainly on the site of tumors. When ACC is located in the upper trachea, tracheal resection may be combined with total laryngectomy and terminal tracheostomy [74]. When ACC involves carina and main bronchi, carina resection and reconstruction are required. A report that analyzed patient survival after ACC complete resection showed comparable five-year survival rates of 52% to 79% and ten-year survival of 29% to 56% [75,76,77,78]. Recurrences after surgery are not uncommon and are mainly due to the ability of the tumor to spread along perineural and lymphatic layers. The role of post-operative radiotherapy in ACC is debated and is generally recommended in the case of incomplete resection [78]. However, despite the lack of a randomized control trial, the available data suggest that adjuvant radiotherapy may delay or even reduce the incidence of local recurrence [78]. Radical radiotherapy alone is performed in patients that are not candidates for surgical resection due to local involvement, extension to contiguous organs, or due to the poor clinical condition of the patient. However, ACC is considered a tumor with low radiation sensitivity and requires a dose of 70 Gy (35 fractions over 7 weeks) to obtain local cancer control [1]. Studies focused only on unresectable ACCs showed five-year OS ranging between 17% to 56% after definitive radiotherapy, with significantly worse outcomes when compared to patients that underwent surgery [79,80,81]. However, recent retrospective data showed no significant difference between operated and non-operated patients with ACCs, despite local relapse being observed mainly in non-operated patients [82]. The role of systemic treatments in the management of recurrent or metastatic ACC is not well defined due to the few clinical trials published so far. ACC is considered a histotype that exhibits poor sensitivity to chemotherapy, and it is unlikely that chemotherapy will alter the natural history of the disease. Several single-agent standard cytotoxic drugs were studied in a clinical trial, which enrolled few patients, showing infrequent objective responses [83]. Vinorelbine and epirubicin are probably single-cytotoxic agents of choice since some objective responses have been reported for these agents, while paclitaxel is not recommended based on an Eastern Cooperative Oncology Group (ECOG) phase 2 study [84]. Combination regimens containing cisplatin, generally in conjunction with anthracycline, improve the response rate compared with single agents; however, an evident response rate is noticeable in no more than 33% of patients [83].

#### 2.1.7. Molecular Therapeutic Targets for ACC

Improved understanding of the molecular mechanisms underlying carcinogenesis in ACC has opened up new treatment perspectives, particularly through the potential use of molecular target therapy in patients suffering from locally advanced, recurrent, or distantly metastatic disease. Target therapies against c-KIT receptor, using tyrosine kinase inhibitors, were the first to be explored in clinical trials. In two phase II trials, 26 patients with advanced and metastatic c-Kit-positive ACC were treated with imatinib (400 mg orally bid) without any responses and with disease progression in the majority of subjects within 6 months [85,86]. Ghosal et al. explored the efficacy of combined treatment with imatinib and cisplatin in 28 patients with advanced and metastatic ACC with an overexpression of c-KIT. Only 3 of 28 patients showed partial response on subsequent radiological imaging, and the median time to progression and overall survival was 15 months (range 1–43) and 35 months (range 1–75), respectively [87]. Wong at al. conducted a phase II trial treating patients with recurrent/metastatic ACC, with c-KIT overexpression, with desatinib (70 mg orally bid). The response was assessed every eight weeks using RECIST [88]. However, only one objective response (2.5%) was reported, and median survival was 14.5 months [88]. Chau et al. conducted a single-arm, phase II trial that enrolled fourteen patients suffering from recurrent and/or metastatic ACC, who were treated with sunitinib (37.5 mg daily), a multi-targeted inhibitor of VEGFR, c-KIT, PGFR, ret proto-oncogene (RET) and FMS-like tyrosine kinase 3 (FLT3). No objective responses were observed and median overall survival was 18.7 months. Furthermore, toxic effects occur in at least 50% of patients, including fatigue, oral mucositis and hypophosphatemia [89]. Based on the results of these studies, we can conclude that the inhibition of the c-KIT pathway is not able to modify the natural history of the ACC disease. Target EGFR pathway is another potential therapeutic strategy in ACC. Unfortunately, some trials that used different agents such as gefitinib, cetuximab and lapatinib have shown no objective responses in previously treated patients [90,91,92]. Fibroblast growth factor receptor 1 (FGFR1) is a downstream pathway of the MYB gene; therefore, target FGFR1 could inhibit one of the main molecular dysregulation pathways in the pathogenesis of ACC. In a phase II study, 35 patients with progressive ACC were treated with dovitinib, a small molecular inhibitor of FGFR1, and it was found that 6% of patients showed a partial response while 65% exhibited stable disease; however, progression-free survival (8.2 months) was more favorable when compared with other molecular target agents [93]. Lenvatinib is a multi-kinase inhibitor against FGFR1, VEGFR2, c-KIT, RET, PDFR-α and PDGFR-β. Locati et al. enrolled twenty-eight patients with recurrent/metastatic ACC who were treated with oral lenvatinib at a dose of 24 mg/day [94]. Here, three partial responses (11.5%) were reported; however, target lesion reductions between 23% and 28% were observed in 4 of 20 patients with stable disease. The median overall survival and progression-free survival were 27 months and 9.1 months, respectively. Treatment-related adverse events were frequent (fatigue, dry mouth); thus, the dose of lenvatinib was reduced in 24 patients [94]. Similarly, Tchekmedyian et al. treated 32 patients suffering from recurrent/metastatic ACC with lenvatinib 24 mg orally per day, and reported a partial response rate of 15.6% [95]. However, eight patients (25%) showed more than 20% reduction in tumor size. The median progression-free survival time was 17.5 months. A total of 23 patients required at least one dose modification, and 18 of 32 patients discontinued Lenvatinib for drug-related side effects. The most common grade 3 adverse events were hypertension and oral pain, but three grade 4 adverse events were observed (myocardial infarction, posterior reversible encephalopathy syndrome and intracranial hemorrhage) [95]. These results show that Lenvatinib has an anti-cancer effect despite toxicity being common and dose reduction being necessary for 72% to 86% of patients. However, Lenvatinib was designed as a National Comprehensive Cancer Network (NCCN) grade 2b recommendation for treatment of progressive/metastatic ACC in the NCCH Head and Neck Cancer guidelines [96]. Several inhibitors of VEGFR pathways, such as sorafenib, regorafenib, axitinib and pazopanib, have been studied in ACC and have shown some clinical benefit, with the overall response rate ranging between 0% and 10%, and prolonged stable disease (>6 months) in up to 85% of unselected patients [97,98,99,100]. The NOTCH signaling pathway is a well-known critical regulator of cell proliferation and seems to play a significant role in 20% of recurrent/metastatic ACC due to the evidence of NOTCH-activating mutations; therefore, the inhibition of this pathway could be a favorable molecular target in aggressive phenotypes of ACC. However, preliminary results of a human phase I trial with the pan-notch inhibitor LY3039478 demonstrated only minimal responses, with a partial response in 1 out of 22 unselected patients, while 4 patients showed stable disease for over 6 months [101]. Treatment-related adverse events included diarrhea, fatigue, vomiting, dry mouth and dry skin [101]. Brontictuzumab is a monoclonal antibody that targets Notch1, binding the juxtamembrane negative regulatory region, and inhibits pathway activation. Ferrarotto et al. conducted a phase I study treating 48 patients suffering from refractory solid cancers with brontictuzmab intravenously at various dose levels [102]. Clinical benefit was seen in 6 of 36 (17%) assessable subjects, among which 5 were affected by ACC with evidence of Notch1 pathway activation, 2 with partial response and 3 prolonged disease stabilization (>6 months) [102]. CB-103 is an upstream inhibitor of the NOTCH pathway that in a phase 1 trial showed a median progression-free survival of 22 weeks in ACC patients. No discontinuations occurred due to treatment-related adverse events [103]. AL101 is a small molecule that inhibits gamma-secretase, an enzyme that plays a key role in the activation of the Notch pathway by releasing the NICD of Notch receptors. The preliminary data from the ACCURACY trial, a phase 2, open-label, multi-center study of AL101 in patients with ACC-bearing activating NOTCH mutations, showed a response rate of 15%, confirming the potential anti-tumor activity of Notch pathway inhibition [104]. Another potential molecular target in the treatment of ACC is the PI3K/PTEN/mTOR pathway. Everolimus was tested for progressive unresectable ACC patients in a phase 2 trial. No patients achieved a partial response by RECIST; however, 44% of 18 patients with available data about pre-treatment and post-treatment positron emission tomography-CT scan showed a metabolic partial response, defined as a >25% reduction in maximum standardized uptake values (SUVmax). Furthermore, metabolic partial response was associated with longer median progression-free survival [105]. Despite the poor representation of PDL-1 expression and the low immunogenicity of the ACC microenvironment, some trials have tested checkpoint inhibitor therapy with limited success. The NISCAHN trial evaluated the efficacy of nivolumab in the treatment of 45 recurrent metastatic ACC patients. An 8.7% partial response and 56% of stable disease were observed in ACC patients, while the median progressive-free survival was 5 months [106]. Pembrolizumab was tested for salivary gland carcinomas as a single agent and in association with hypo-fractionate radiotherapy in advanced ACCs, but no evidence of the tumor response was highlighted [107,108]. Table 1 summarizes the studies on molecular therapeutic targets for ACC.

### 2.2. Mucoepidermoid Carcinoma of the Trachea

Mucoepidermoid carcinoma (MEC) is a rare type of salivary gland cancer that can originate in the upper airways [1]. Most commonly, it arises in the posterior wall of the trachea, and histology displays cellular heterogeneity containing various proportions of mucin-secreting cells, epidermoid cells and cells of intermediate type, with varying architectural formations from cystic structures to small solid nests or glandular-like structures. MEC is classified as high-grade or low-grade based on the histologic appearance, local invasion, cellular atypia and presence of necrosis. A recent retrospective study suggests that MEC with airway involvement has different biological behaviors and better prognosis than ACC. However, MEC patients showed more bronchial tumors when compared to ACC; therefore, these results may not be generalizable to tumors confined to the trachea [109]. Furthermore, the clinical course of these tumors is correlated with the histological grade; thus, high-grade-MEC of the trachea generally carries a worse prognosis. The preferred treatments in the curative-intent setting include tracheal surgery and adjuvant radiotherapy. However, therapies for relapsed/metastatic or unresectable disease are notoriously ineffective; thus, new targets are required for precision therapy in patients suffering from MEC. A recurrent t(11;19)(q14–21;p12–13) translocation encoding a potentially novel CRTC1-MAML2 gene fusion has been detected in up to 80% of MEC cases, and this biological feature is occasionally the sole cytogenetic alteration [110]. The CRTC1-MAML2 fusion protein consists of the 42-aa CREB binding domain (CBD) of the CREB transcriptional coactivator CRTC1 at its N terminus and the 981-aa transcriptional activation domain (TAD) of the Notch transcriptional co-activator MAML2 at its C terminus [111]. Studies in vitro showed that CRTC1-MAML2 constitutes an oncogenic driver in MEC development since MEC cells depend on its expression for growth and survival [112]. CRTC1-MAML2 fusion acts through an interaction with CREB, resulting in aberrant activation of the CREB-mediated transcriptional program; furthermore, fusion protein is also able to interact with and activate MYC and AP-1 [113,114,115]. Ni et al. have shown that the CRTC1-MAML2 fusion upregulates the expression of amphiregulin (AREG), an EGFR ligand, resulting in EGFR pathway activation in an autocrine manner [116]. MEC cells bearing the fusion gene have shown high sensitivity to EGFR signal inhibition through the use of the EGFR monoclonal antibody Cetuximab, with an evident inhibition effect on cell growth in vitro and in vivo [116]. However, EGFR inhibition is unable to eradicate all the MEC cells, and usually, a proportion of the surviving cells may develop resistance associated with anti-EGFR therapies in a clinical setting. Some evidence suggests that MEC arises from the transformation of salivary gland stem/progenitor cells and is maintained by MEC stem-like cells regulated through Notch signaling [116]. Cancer stem-like cells constitute a subpopulation of highly tumorigenic cells that in different cancers have shown a key role in tumor maintenance and metastatic ability [117]. Given the essential role of Notch signaling in maintaining the MEC stem-cell-like compartment, targeting Notch could effectively contribute to control MEC recurrence due to the action of the cancer stem-cell-like compartment. Furthermore, data extrapolated from an in vitro study suggest that treatments co-targeting Notch and EGFR signaling could be promising as anti-MEC treatment [116]. Wang et al. used comprehensive genomic profiling (CGP) to describe the genomic landscape of a large group of clinically advanced MECs, identifying recurring genomic alterations [118]. MECs showed a similar number of genetic alterations per tumor to adenocarcinomas but a greater number than ACC. TP53 mutations and cyclin family genetic alterations were common while ERBB2 amplification was uncommon in MECs, occurring in less than 10% of tumors. High-grade MEC showed greater genetic complexity compared to low- and intermediate-grade tumors, with a high frequency of PI3K pathway activation, suggesting a potential role of PI3K/mTOR inhibitors [118]. Alteration in DNA repair genes such as BAP1 (BRCA1-associated protein 1) was detected in 10% of tumors, while 10% harbored BRCA gene alterations. BAP1 is a nuclear-localized deubiquitinating enzyme able to regulate transcription, cell cycling and DNA damage repair, which is commonly altered in sporadic melanoma, renal cell carcinoma and mesothelioma. Clinical trials focused on MEC target therapy are lacking; however, some patients affected by MEC were enrolled in clinical trials that evaluated the effect of different agents on patients suffering from recurrent/metastatic salivary gland tumors. In a phase 2 trial, sorafenib showed in two MEC patients, with high expression of VEGF and ANG2, the ability to decrease disease progression, probably through antiangiogenetic activity [119]. Tumor necrosis with cavitation occurred in one patient with a high-grade MEC [119]. Nintedanib was tested in a population of patients with different subtypes of salivary gland cancers, among which two patients (10%) were affected by MEC. However, there were no partial responders, although 75% of patients achieved disease stabilization with a median duration of 8.2 months (range, 1.76–12.36 months) [120]. In a phase 2 study, lapatinib showed no objective response in two MEC patients, but stable disease (>6 months) was observed in 36% of all the eligible patients [92].

### 2.3. Primitive Squamous Cell Carcinoma of the Trachea

Squamous cell carcinoma (SCC) is the most frequent histological type of primary tumor arising from the trachea [1]. Patients with SCC, unlike patients affected by ACC or MEC, commonly have a history of smoking [1]. Although a delay in diagnosis is a typical feature of all primary tumors of the trachea, the duration of symptoms prior to diagnosis is shorter in SCC than in ACC [121]. Furthermore, 60% of patients suffering from SCC present with hemoptysis, compared to 30% of patients with ACC [1]. Surgical resection and adjuvant radiotherapy are treatments of choice. However, tumor extension, lymphatic invasion, mediastinal extension or distant metastases all are factors that may limit surgical resection. In patients with SCC who underwent resection, the overall 5- and 10-year survival was 39% and 18%, compared to 52% and 29%, respectively in patients with ACC who underwent resection [76]. In the Epidemiology and End Result program (SEER) database of 578 cases of primary tracheal tumors, the overall 5-year survival was better in ACC (74%) than in SCC (12.6%), underlining the different biological behavior of the two main tracheal tumor histotypes [122]. However, in a retrospective matched-pair analysis of the SEER database, treatment with radiation was associated with improved survival, particularly in patients with SCC [123]. The biological features and histologic appearance of the SCC of the trachea are identical to the SCC of the lung; thus, molecular alterations and the response to treatment of the most studied lung cancer could be translated. Therefore, patients suffering from unresectable/metastatic SCC of the trachea should be managed with the same treatments that are used in the squamous histotype of the lung. Unlike salivary gland tumors, immune checkpoint inhibition has opened the door to new treatment options for patients with SCC of the lung. KEYNOTE-024 established the use of pembrolizumab monotherapy as the standard of care for patients suffering from metastatic non-small lung cancer with 50% or greater tumoral PD-L1 expression [124]. Among the patients enrolled in this trial, 18% had SCC. The primary outcome was progression-free survival, which was 10.3 months for pembrolizumab versus 6.0 months for chemotherapy (hazard ratio 0.50; 95% CI, 0.37–0.68; *p* < 0.001). The objective response rate for patients treated with pembrolizumab was 44.8% versus 27.8% for patients treated with chemotherapy. Furthermore, pembrolizumab resulted in a significant survival benefit in all patient subgroups, including SCC. However, in patients affected by SCC with PD-L1 expression less than 50%, pembrolizumab showed no additional benefit compared to platinum doublet chemotherapy [125]. Based on KEYNOTE-407 results, platinum doublet chemotherapy plus immunotherapy has been recently approved for use as first-line therapy in patients with SCC of the lung regardless of the level of PD-L1 expression. The addition of pembrolizumab to chemotherapy with carboplatin plus paclitaxel or nab-paclitaxel resulted in significantly longer overall survival and progression-free survival compared to chemotherapy alone [126]. Recent comprehensive genomic analysis has defined the genomic and epigenomic alterations driving lung SCC, with the identification of relatively high-frequency recurrent somatic alteration. The most common genetic alterations are the loss of TP53 and CDKN2A [127]. However, other highly prevalent genetic alterations that occur in a mutually exclusive manner are the alteration of the nuclear factor erythroid 2-like 2 (NFE2L2)/kelch-like ECH-associated protein 1 (KEAP1)/cullin 3 (CUL3) pathway (i.e., mutations of NFE2L2 or KEAP1), which regulates the response to oxidative stress, and the truncating mutations of the NOTCH1 gene [128]. A common alteration described in SCC of different organs is the amplification of 3q, a region containing SOX, TP63 and PIK3CA, but also the amplification of 7p11 and 8p12, regions where the EGFR and FGFR1 genes are located [128]. However, despite EGFR amplification occurring in 7% of lung SCC cases, EGFR exon deletions and exon 21 L858R mutations are absent [128]. Furthermore, KRAS mutations are a rare occurrence in SCC. Considering the frequent focal amplification of FGFR1 and the recurrent activating mutations of FGF2 and FGF3, the fibroblast growth factor receptor family may represent one of the main potential targets in the treatment of advanced/metastatic SCC. However, initial clinical data suggest that only a limited number of SCC patients with FGFR1 amplification may have a clinical benefit from FGFR kinase inhibitors [129,130]. Another potential target in SCC molecular therapy involves members of the PIK3 pathway. PIK3CA mutations/amplification and PTEN loss are not uncommon in SCC and are sensitive to PI3K inhibitors [131]. Unfortunately, early phase studies evaluating treatment with PI3K inhibitors found no significant clinical benefit from the treatment, with a response rate that does not exceed 5% and medial progressive-free survival similar to what was obtained from docetaxel [132,133,134].

## 3. Conclusions

Primitive tracheal tumors are rare and are typically treated with surgery and adjuvant radiotherapy [135]. However, surgery can be limited by several factors such as the excessive extent of tracheal involvement by the tumor, a poor patient clinical condition, contiguous organ infiltrations, and the presence of distant metastasis [136]. Patients suffering from unresectable/recurrent or metastatic disease have limited therapeutic options. RT may provide survival benefits for tracheal cancer patients who do not undergo surgery, while CT generally does not allow further outcome improvement [137]. However, tumors arising from the salivary glands exhibit poor sensitivity to chemotherapy and/or radiotherapy. Nevertheless, the improvement and the deeper knowledge of the genetic and biological alterations of these different types of tumors may precisely characterize the molecular profile of each patient, opening up new targeted treatment perspectives. Several targets are under clinical investigation, and improvements in target therapy against driver mutations will be the starting point for the next trials involving patients affected by this rare disease.

Finally, improving early diagnosis may improve the standard of living and the survival rate since delayed diagnosis and treatment may impair patient survival.

## Figures and Tables

**Table 1:** Published studies with available data on molecular therapeutic targets for ACC. ORR: objective response rate, mPFS: median progression-free survival, NR: not reached.

Study	Number of Treated Patients	Subtype	Drug	ORR(%)	mPFS (mo)
Gilbert et al. [84]	45	Salivary gland carcinoma	Paclitaxel	0	4
Pfeffer et al. [85]	10	Adenoid cystic carcinoma	Imatinib	0	6
Hotte et al. [86]	15	Adenoid cystic carcinoma	Imatinib	0	2.5
Ghosal et al. [87]	28	Adenoid cystic carcinoma	Imatinib + Cisplatin	0	15
Wong et al. [88]	40	Adenoid cystic carcinoma	Desatinib	2.5	4.8
Chau et al. [89]	13	Adenoid cystic carcinoma	Sunitinib	0	7.2
Jakob et al. [90]	19	Adenoid cystic carcinoma	Gefitinib	0	4.3
Locati et al. [91]	30	Adenoid cystic + non adenoid cystic carcinoma	Cetuximab	0	6
Agulnik et al. [92]	19	Adenoid cystic carcinoma	Lapatinib	0	3.5
Dillon et al. [93]	34	Adenoid cystic carcinoma	Dovitinib	0	8.2
Locati et al. [94]	26	Adenoid cystic carcinoma	Lenvatinib	0	9.1
Tchekmedyian et al. [95]	32	Adenoid cystic carcinoma	Lenvatinib	0	17.5
Pfister et al. [96]	32	Adenoid cystic carcinoma	Lenvatinib	15.6	17.5
Ho et al. [97]	33	Adenoid cystic carcinoma	Axitinib	0	5.7
Ho et al. [98]	38	Adenoid cystic carcinoma	Regorafenib	NR	NR
Thomson et al. [99]	23	Adenoid cystic carcinoma	Sorafenib	0	11.3
Guigay et al. [100]	46	Adenoid cystic carcinoma	Pazopanib	0	5.9
Even et al. [101]	22	Adenoid cystic carcinoma	NOTCH inhibitor crenigacestat (LY3039478)	NR	5.3
Ferrarotto et.al. [102]	48	Solid tumors (adenoid cystic carcinoma)	Brontictuzumab	NR	2
Miranda et al. [103]	41	Solid tumors and hematological malignancies (adenoid cystic carcinoma)	CB-103	NR	5
ACCURACY [104]	87	Adenoid cystic carcinoma	AL 101	15	NR
Dong-Wan et al. [105]	34	Adenoid cystic carcinoma	Everolimus	NR	11.2
Fayette et al. NISCAHN. [106]	46	Salivary gland carcinoma	Nivolumab	0	4.9
KEYNOTE-028 [107]	26	Salivary gland carcinoma	Pembrolizumab	12	4
Mahmood et al. [108]	10	Adenoid cystic carcinoma	Pembrolizumab	0	4.5

## Data Availability

Not applicable.

## Abbreviations

AHRF	acute hypoxic respiratory failure
ACC	adenoid cystic carcinoma
COPD	obstructive pulmonary disease
FISH	fluorescent in situ hybridization
Cdks	cyclin-dependent kinase
NFIB	Nuclear Factor I B
CCNBI	Cyclin B1
MAD1L1	Mitotic Arrest Deficient 1 Like 1
BIRC3	Baculoviral IAP Repeat Containing 3
HSPA8	Heat Shock Protein Family A (Hsp70) Member 8
MYC	MYC Proto-Oncogene, BHLH Transcription Factor
VEGFA	Vascular Endothelial Growth Factor A
FGF2	Fibroblast Growth Factor 2
SEMA4D	Semaphorin 4D
ATR	ataxia telangiectasia and Rad3-related (ATR)
VX970	Berzosertib
siRNA	Short Interfering RNA
CDH1	Cadherin 1
CDH2	Cadherin 2
VIM	Vimentin
ACT2	Actin Alpha 2, Smooth Muscle
EMT	epithelial–mesenchymal transition
MCL-1	myeloid cell leukemia-1
BAX	BCL-2-associated X protein
MMP7	Matrix Metallopeptidase 7
MMP9	Matrix Metallopeptidase 9
ICAM-I	Intercellular Adhesion Molecule 1
SCF	Stem Cell Factor
GIST	gastrointestinal stromal tumor
MAPK	mitogen activated protein kinase
ERK	extracellular signal-regulated kinase
PI3K/AKT	phosphatidylinositol 3kinase/protein kinase B
PLC-γ	phospholipase-C-γ
FISH	fluorescence in situ hybridization
EGFR	Epidermal growth factor receptor
KRAS	Kristen rat sarcoma viral oncogene homologue
VEGF	Vascular Endothelial Growth Factor
DLL1	Delta Like Canonical Notch Ligand 1
JAG1	Jagged 1
NECD	Notch extracellular domain
MAML	Mastermind-like
PTEN	Phosphatase and tensin homolog
PI3K	phosphatidylinositol 3-kinase
TIL	tumor-infiltrating lymphocytes
Grb	granzyme B
DC	Dendritic cells
PD-1	Programmed Cell Death protein 1
RGMb	Repulsive Guidance Molecule B
FLT3	FMS-like tyrosine kinase 3
FGFR1	Fibroblast growth factor receptor 1
NCCN	National Comprehensive Cancer Network
TP53	Tumor Protein P53
NFE2L2	nuclear factor erythroid 2-like 2
CUL3	cullin 3

## References

[1] Macchiarini P. (2006). Primary tracheal tumours. Lancet Oncol..

[2] Junker K. (2014). Pathology of Tracheal Tumors. Thorac. Surg. Clin..

[3] Moores D., Mane P. (2018). Pathology of Primary Tracheobronchial Malignancies Other than Adenoid Cystic Carcinomas. Thorac. Surg. Clin..

[4] Gaissert H.A., Honings J., Gokhale M. (2009). Treatment of Tracheal Tumors. Semin. Thorac. Cardiovasc. Surg..

[5] Maziak D.E. (2018). Biology of Adenoid Cystic Carcinoma of the Tracheobronchial Tree and Principles of Management. Thorac. Surg. Clin..

[6] Billroth T. (1859). Beobachtungen űber geschwűlsteder Speicheldrűsen. Virchous Arch. Pathol. Anat..

[7] Jaso J., Malhotra R. (2011). Adenoid cystic carcinoma. Arch. Pathol. Lab. Med..

[8] Szanto P.A., Luna M.A., Tortoledo M.E., White R.A. (1984). Histologic grading of adenoid cystic carcinoma of the salivary glands. Cancer.

[9] da Cruz Perez D.E., de Abreu Alves F., Nobuko Nishimoto I., de Almeida O.P., Kowalski L.P. (2006). Prognostic factors in head and neck adenoid cystic carcinoma. Oral Oncol..

[10] Fordice J., Kershaw C., El-Naggar A., Goepfert H. (1999). Adenoid cystic carcinoma of the head and neck: Predictors of morbidity and mortality. Arch. Otolaryngol. Head Neck Surg..

[11] Cheuk W., Chan J.K., Ngan R.K. (1999). Dedifferentiation in adenoid cystic carcinoma of salivary gland: An uncommon complication associated with an accelerated clinical course. Am. J. Surg. Pathol..

[12] Meis J.M. (1991). “Dedifferentiation” in bone and soft-tissue tumors. A histological indicator of tumor progression. Pathol. Annu..

[13] Seethala R.R., Hunt J.L., Baloch Z.W., Livolsi V.A., Leon Barnes E. (2007). Adenoid cystic carcinoma with high-grade transformation: A report of 11 cases and a review of the literature. Am. J. Surg. Pathol..

[14] Chau Y.-P., Hongyo T., Aozasa K., Chan J.K. (2001). Dedifferentiation of adenoid cystic carcinoma: Report of a case implicating p53 gene mutation. Hum. Pathol..

[15] Sandros J., Stenman G., Mark J. (1990). Cytogenetic and molecular observations in human and experimental salivary gland tumors. Cancer Genet. Cytogenet..

[16] Persson M., Andren Y., Mark J., Horlings H.M., Persson F., Stenman G. (2009). Recurrent fusion of MYB and NFIB transcription factor genes in carcinomas of the breast and head and neck. Proc. Natl. Acad. Sci. USA.

[17] Mucenski M.L., McLain K., Kier A.B., Swerdlow S.H., Schreiner C.M., Miller T.A., Pietryga D.W., Scott W.J., Potter S. (1991). A functional c-myb gene is required for normal murine fetal hepatic hematopoiesis. Cell.

[18] Jiang J., Best S., Menzel S., Silver N., Lai M.I., Surdulescu G.L., Spector T.D., Thein S.L. (2006). cMYB is involved in the regulation of fetal hemoglobin production in adults. Blood.

[19] Nakata Y., Shetzline S., Sakashita C., Kalota A., Rallapalli R., Rudnick S.I., Zhang Y., Emerson S.G., Gewirtz A.M. (2007). c-Myb contributes to G2/M cell cycle transition in human hematopoietic cells by direct regulation of cyclin B1 expression. Mol. Cell Biol..

[20] Cicirò Y., Sala A. (2021). MYB oncoproteins: Emerging players and potential therapeutic targets in human cancer. Oncogenesis.

[21] Mitani Y., Li J., Rao P.H., Zhao Y.J., Bell D., Lippman S.M., Weber R.S., Caulin C., El-Naggar A.K. (2010). Comprehensive analysis of the MYB-NFIB gene fusion in salivary adenoid cystic carcinoma: Incidence, variability, and clinicopathologic significance. Clin. Cancer Res..

[22] Drier Y., Cotton M.J., Williamson K.E., Gillespie S.M., Ryan R.J.H., Kluk M.J., Carey C.D., Rodig S.J., Sholl L.M., Afrogheh A.H. (2016). An oncogenic MYB feedback loop drives alternate cell fates in adenoid cystic carcinoma. Nat. Genet..

[23] Frerich C.A., Sedam H.N., Kang H., Mitani Y., El-Naggar A.K., Ness S.A. (2019). N-Terminal Truncated Myb with New Transcriptional Activity Produced Through Use of an Alternative MYB Promoter in Salivary Gland Adenoid Cystic Carcinoma. Cancers.

[24] Andersson M.K., Mangiapane G., Nevado P.T., Tsakaneli A., Carlsson T., Corda G., Nieddu V., Abrahamian C., Chayka O., Rai L. (2020). ATR is a MYB regulated gene and potential therapeutic target in adenoid cystic carcinoma. Oncogenesis.

[25] Gaillard H., Garcia-Muse T., Aguilera A. (2015). Replication stress and cancer. Nat. Rev. Cancer.

[26] Murga M., Campaner S., Lopez-Contreras A.J., Toledo L.I., Soria R., Montaña M.F., Artista L.D., Schleker T., Guerra C., Garcia E.O. (2011). Exploiting oncogene-induced replicative stress for the selective killing of Myc-driven tumors. Nat. Struct. Mol. Biol..

[27] Koniaras K., Cuddihy A.R., Christopoulos H., Hogg A., O’Connell M.J. (2001). Inhibition of Chk1-dependent G2 DNA damage checkpoint radiosensitizes p53 mutant human cells. Oncogene.

[28] López-Contreras A.J., Gutierrez-Martinez P., Specks J., Rodrigo-Perez S., Fernandez-Capetillo O. (2012). An extra allele of Chk1 limits oncogene-induced replicative stress and promotes transformation. J. Exp. Med..

[29] Abdel-Fatah T.M., Middleton F.K., Arora A., Agarwal D., Chen T., Moseley P.M., Perry C., Doherty R., Chan S., Green A.R. (2015). Untangling the ATR-CHEK1 network for prognostication, prediction and therapeutic target validation in breast cancer. Mol. Oncol..

[30] Hilton B.A., Li Z., Musich P.R., Wang H., Cartwright B.M., Serrano M., Zhou X.Z., Lu K.P., Zou Y. (2015). ATR Plays a Direct Antiapoptotic Role at Mitochondria, which Is Regulated by Prolyl Isomerase Pin1. Mol. Cell.

[31] Xu L.-H., Zhao F., Yang W.-W., Chen C.-W., Du Z.-H., Fu M., Ge X.-Y., Li S.-L. (2019). MYB promotes the growth and metastasis of salivary adenoid cystic carcinoma. Int. J. Oncol..

[32] Thiery J.P., Acloque H., Huang R.Y.J., Nieto M.A. (2009). Epithelial-Mesenchymal Transitions in Development and Disease. Cell.

[33] Xiang W., Yang C.-Y., Bai L. (2018). MCL-1 inhibition in cancer treatment. OncoTargets Ther..

[34] Akgul C. (2009). Mcl-1 is a potential therapeutic target in multiple types of cancer. Cell Mol. Life Sci..

[35] Da Silva F.J., de Azevedo J.C., Lima Ralph A.C., Viana Pinheiro J.d.J., Morais Freitas V., Queiroz Calcagno D. (2023). Salivary glands adenoid cystic carcinoma: A molecular profile update and potential implications. Front. Oncol..

[36] Babaei M.A., Kamalidehghan B., Saleem M., Huri H.Z., Ahmadipour F. (2016). Receptor tyrosine kinase (c-Kit) inhibitors: A potential therapeutic target in cancer cells. Drug Des. Dev. Ther..

[37] Besmer P., Murphy J.E., George P.C., Qiu F., Bergold P.J., Lederman L., Snyder H.W., Brodeur D., Zuckerman E.E., Hardy W.D. (1986). A new acute transforming feline retrovirus and relationship of its oncogene v-kit with the protein kinase gene family. Nature.

[38] Sheikh E., Tran T., Vranic S., Levy A., Bonfil R.D. (2022). Role and Significance of c-KIT Receptor Tyrosine Kinase in Cancer: A Review. Bosn. J. Basic. Med. Sci..

[39] Levy D.E., Darnell J.E. (2002). STATs: Transcriptional control and biological impact. Nat. Rev. Mol. Cell Biol..

[40] Vila L., Liu H., Al-Quran S.Z., Coco D.P., Dong H.-J., Liu C. (2009). Identification of c-kit gene mutations in primary adenoid cystic carcinoma of the salivary gland. Mod. Pathol..

[41] Jeng Y.-M., Lin C.-Y., Hsu H.-C. (2000). Expression of the c-kit protein is associated with certain subtypes of salivary gland carcinoma. Cancer Lett..

[42] Holst V.A., Marshall C., Moskaluk C.A., Frierson H.F. (1999). KIT protein expression and analysis of c-kit gene mutation in adenoid cystic carcinoma. Mod. Pathol..

[43] Freier K., Flechtenmacher C., Walch A., Devens F., Mühling J., Lichter P., Joos S., Hofele C. (2005). Differential KIT expression in histological subtypes of adenoid cystic carcinoma (ACC) of the salivary gland. Oral. Oncol..

[44] Hajjo R., Sweidan K. (2020). Review on Epidermal Growth Factor Receptor (EGFR) Structure, Signaling Pathways, Interactions, and Recent Updates of EGFR Inhibitors. Curr. Top. Med. Chem..

[45] Singh D., Attri B.K., Gill R.K., Bariwal J. (2016). Review on EGFR Inhibitors: Critical Updates. Mini Rev. Med. Chem..

[46] Dahse R., Driemel O., Schwarz S., Kromeyer-Hauschild K., Berndt A., Kosmehl H. (2009). KRAS status and epidermal growth factor receptor expression as determinants for anti-EGFR therapies in salivary gland carcinomas. Oral. Oncol..

[47] Saida K., Murase T., Ito M., Fujii K., Takino H., Masaki A., Kawakita D., Ijichi K., Tada Y., Kusafuka K. (2018). Mutation analysis of the EGFR pathway genes, *EGFR*, *RAS*, *PIK3CA*, *BRAF*, and *AKT1*, in salivary gland adenoid cystic carcinoma. Oncotarget.

[48] Benesova L., Minarik M., Jancarikova D., Belsanova B., Pesek M. (2010). Multiplicity of EGFR and KRAS mutations in non-small cell lung cancer (NSCLC) patients treated with tyrosine kinase inhibitors. Anticancer. Res..

[49] Huang L., Guo Z., Wang F., Fu L. (2021). KRAS mutation: From undruggable to druggable in cancer. Signal Transduct. Target. Ther..

[50] Miller L.E., Au V., Mokhtari T.E., Goss D., Faden D.L., Varvares M.A. (2022). A Contemporary Review of Molecular Therapeutic Targets for Adenoid Cystic Carcinoma. Cancers.

[51] Siveen K.S., Prabhu K., Krishnankutty R., Kuttikrishnan S., Tsakou M., Alali F., Dermime S., Mohammad R.M., Uddin S. (2017). Vascular Endothelial Growth Factor (VEGF) Signaling in Tumour Vascularization: Potential and Challenges. Curr. Vasc. Pharmacol..

[52] Li Z., Tang P., Xu Z. (2001). Clinico-pathological significance of microvessel density and vascular endothelial growth factor expression in adenoid cystic carcinoma of salivary glands. Zhonghua Kou Qiang Yi Xue Za Zhi.

[53] Park S., Nam S.J., Keam B., Kim T.M., Jeon Y.K., Lee S.-H., Hah J.H., Kwon T.-K., Kim D.-W., Sung M.-W. (2016). VEGF and Ki-67 Overexpression in Predicting Poor Overall Survival in Adenoid Cystic Carcinoma. Cancer Res. Treat..

[54] Lobry C., Oh P., Mansour M., Look A.T., Aifantis I. (2014). Notch signaling: Switching an oncogene to a tumor suppressor. Blood.

[55] Gragnani L., Lorini S., Marri S., Zignego A.L. (2020). Role of Notch Receptors in Hematologic Malignancies. Cells.

[56] Parmigiani E., Taylor V., Giachino C. (2020). Oncogenic and Tumor-Suppressive Functions of NOTCH Signaling in Glioma. Cells.

[57] Chanrion M., Kuperstein I., Barrière C., El Marjou F., Cohen D., Vignjevic D., Stimmer L., Paul-Gilloteaux P., Bieche I., Tavares S.D.R. (2014). Concomitant Notch activation and p53 deletion trigger epithelial-to-mesenchymal transition and metastasis in mouse gut. Nat. Commun..

[58] Xie M., Zhang L., He C.-S., Xu F., Liu J.-L., Hu Z.-H., Zhao L.-P., Tian Y. (2012). Activation of Notch-1 enhances epithelial-mesenchymal transition in gefitinib-acquired resistant lung cancer cells. J. Cell Biochem..

[59] Ferrarotto R., Mitani Y., Diao L., Guijarro I., Wang J., Zweidler-McKay P., Bell D., William W.N., Glisson B.S., Wick M.J. (2017). Activating NOTCH1 Mutations Define a Distinct Subgroup of Patients With Adenoid Cystic Carcinoma Who Have Poor Prognosis, Propensity to Bone and Liver Metastasis, and Potential Responsiveness to Notch1 Inhibitors. J. Clin. Oncol..

[60] Chintakuntlawar A.V., Okuno S.H., Price K.A.R. (2015). Genomic testing may offer therapeutic opportunity in salivary gland cancers. J. Clin. Oncol..

[61] Su B.-H., Qu J., Song M., Huang X.-Y., Hu X.-M., Xie J., Zhao Y., Ding L.-C., She L., Chen J. (2014). NOTCH1 signaling contributes to cell growth, anti-apoptosis and metastasis in salivary adenoid cystic carcinoma. Oncotarget.

[62] Fusco N., Sajjadi E., Venetis K., Gaudioso G., Lopez G., Corti C., Rocco E.G., Criscitiello C., Malapelle U., Invernizzi M. (2020). PTEN Alterations and Their Role in Cancer Management: Are We Making Headway on Precision Medicine?. Genes.

[63] Liu H., Du L., Wang R., Wei C., Liu B., Zhu L., Liu P., Liu Q., Li J., Lu S.-L. (2015). High frequency of loss of PTEN expression in human solid salivary adenoid cystic carcinoma and its implication for targeted therapy. Oncotarget.

[64] Ho A.S., Kannan K., Roy D.M., Morris L.G.T., Ganly I., Katabi N., Ramaswami D., Walsh L.A., Eng S., Huse J.T. (2013). The mutational landscape of adenoid cystic carcinoma. Nat. Genet..

[65] Han Y., Liu D., Li L. (2020). PD-1/PD-L1 pathway: Current researches in cancer. Am. J. Cancer Res..

[66] Ljunggren H.-G., Jonsson R., Höglund P. (2018). Seminal immunologic discoveries with direct clinical implications: The 2018 Nobel Prize in Physiology or Medicine honours discoveries in cancer immunotherapy. Scand. J. Immunol..

[67] Tang Q., Chen Y., Li X., Long S., Shi Y., Yu Y., Wu W., Han L., Wang S. (2022). The role of PD-1/PD-L1 and application of immune-checkpoint inhibitors in human cancers. Front. Immunol..

[68] Mosconi C., de Arruda J.A.A., de Farias A.C.R., Oliveira G.A.Q., de Paula H.M., Fonseca F., Mesquita R.A., Silva T.A., Mendonça E.F., Batista A.C. (2019). Immune microenvironment and evasion mechanisms in adenoid cystic carcinomas of salivary glands. Oral. Oncol..

[69] Teng M.W., Ngiow S.F., Ribas A., Smyth M.J. (2015). Classifying Cancers Based on T-cell Infiltration and PD-L1. Cancer Res..

[70] Sridharan V., Gjini E., Liao X., Chau N.G., Haddad R.I., Severgnini M., Hammerman P., El-Naggar A., Freeman G.J., Hodi F.S. (2016). Immune Profiling of Adenoid Cystic Carcinoma: PD-L2 Expression and Associations with Tumor-Infiltrating Lymphocytes. Cancer Immunol. Res..

[71] Latchman Y., Wood C.R., Chernova T., Chaudhary D., Borde M., Chernova I., Iwai Y., Long A.J., Brown J.A., Nunes R. (2001). PD-L2 is a second ligand for PD-1 and inhibits T cell activation. Nat. Immunol..

[72] Xiao Y., Yu S., Zhu B., Bedoret D., Bu X., Francisco L.M., Hua P., Duke-Cohan J.S., Umetsu D.T., Sharpe A.H. (2014). RGMb is a novel binding partner for PD-L2 and its engagement with PD-L2 promotes respiratory tolerance. J. Exp. Med..

[73] Tapias L.F., Shih A., Mino-Kenudson M., Muniappan A., A Gaissert H., Lanuti M., Mathisen D.J., Ott H.C. (2019). Programmed death ligand 1 and CD8+ immune cell infiltrates in resected primary tracheal malignant neoplasms. Eur. J. Cardio-Thorac. Surg..

[74] Suzuki T. (2011). What is the best management strategy for adenoid cystic carcinoma of the trachea?. Ann. Thorac. Cardiovasc. Surg..

[75] Grillo H.C., Mathisen D.J. (1990). Primary tracheal tumors: Treatment and results. Ann. Thorac. Surg..

[76] Gaissert H.A., Grillo H.C., Shadmehr M., Wright C.D., Gokhale M., Wain J.C., Mathisen D.J. (2004). Long-Term Survival after Resection of Primary Adenoid Cystic and Squamous Cell Carcinoma of the Trachea and Carina. Ann. Thorac. Surg..

[77] Regnard J., Fourquier P., Levasseur P. (1996). Results and prognostic factors in resections of primary tracheal tumors: A multicenter retrospective study. J. Thorac. Cardiovasc. Surg..

[78] Maziak D.E., Todd T.R., Keshavjee S.H., Winton T.L., Van Nostrand P., Pearson F. (1996). Adenoid cystic carcinoma of the airway: Thirty-two-year experience. J. Thorac. Cardiovasc. Surg..

[79] Mendenhall W.M., Morris C.G., Amdur R.J., Werning J.W., Hinerman R.W., Villaret D.B. (2004). Radiotherapy alone or combined with surgery for adenoid cystic carcinoma of the head and neck. Head Neck.

[80] van Weert S., Bloemena E., van der Waal I., de Bree R., Rietveld D.H., Kuik J.D., Leemans C.R. (2013). Adenoid cystic carcinoma of the head and neck: A single-center analysis of 105 consecutive cases over a 30-year period. Oral. Oncol..

[81] Le Péchoux C., Baldeyrou P., Ferreira I., Mahé M. (2005). Cylindromes thoraciques: Thoracic Adenoid cystic carcinomas. Cancer Radiother..

[82] Högerle B.A., Lasitschka F., Muley T., Bougatf N., Herfarth K., Adeberg S., Eichhorn M., Debus J., Winter H., Rieken S. (2019). Primary adenoid cystic carcinoma of the trachea: Clinical outcome of 38 patients after interdisciplinary treatment in a single institution. Radiat. Oncol..

[83] Laurie S.A., Ho A.L., Fury M.G., Sherman E., Pfister D.G. (2011). Systemic therapy in the management of metastatic or locally recurrent adenoid cystic carcinoma of the salivary glands: A systematic review. Lancet Oncol..

[84] Gilbert J., Li Y., Pinto H.A., Jennings T., Kies M.S., Silverman P., Forastiere A.A. (2006). Phase II trial of taxol in salivary gland malignancies (E1394): A trial of the Eastern Cooperative Oncology Group. Head Neck.

[85] Pfeffer M.R., Talmi Y., Catane R., Symon Z., Yosepovitch A., Levitt M. (2007). A phase II study of Imatinib for advanced adenoid cystic carcinoma of head and neck salivary glands. Oral. Oncol..

[86] Hotte S.J., Winquist E.W., Lamont E., MacKenzie M., Vokes E., Chen E.X., Brown S., Pond G.R., Murgo A., Siu L.L. (2005). Imatinib Mesylate in Patients with Adenoid Cystic Cancers of the Salivary Glands Expressing c-kit: A Princess Margaret Hospital Phase II Consortium Study. J. Clin. Oncol..

[87] Ghosal N., Mais K., Shenjere P., Julyan P., Hastings D., Ward T., Ryder W., Bruce I., Homer J., Slevin N. (2011). Phase II study of cisplatin and imatinib in advanced salivary adenoid cystic carcinoma. Br. J. Oral. Maxillofac. Surg..

[88] Wong S., Karrison T., Hayes D., Kies M., Cullen K., Tanvetyanon T., Argiris A., Takebe N., Lim D., Saba N. (2016). Phase II trial of dasatinib for recurrent or metastatic c-KIT expressing adenoid cystic carcinoma and for nonadenoid cystic malignant salivary tumors. Ann. Oncol..

[89] Chau N.G., Hotte S.J., Chen E.X., Chin S.F., Turner S., Wang L., Siu L.L. (2012). A phase II study of sunitinib in recurrent and/or metastatic adenoid cystic carcinoma (ACC) of the salivary glands: Current progress and challenges in evaluating molecularly targeted agents in ACC. Ann. Oncol..

[90] Jakob J.A., Kies M.S., Glisson B.S., Kupferman M.E., Liu D.D., Lee J.J., El-Naggar A.K., Gonzalez-Angulo A.M., Blumenschein G.R. (2015). Phase II study of gefitinib in patients with advanced salivary gland cancers. Head Neck.

[91] Locati L., Bossi P., Perrone F., Potepan P., Crippa F., Mariani L., Casieri P., Orsenigo M., Losa M., Bergamini C. (2009). Cetuximab in recurrent and/or metastatic salivary gland carcinomas: A phase II study. Oral. Oncol..

[92] Agulnik M., Cohen E.W., Cohen R.B., Chen E.X., Vokes E.E., Hotte S.J., Winquist E., Laurie S., Hayes D.N., Dancey J.E. (2007). Phase II Study of Lapatinib in Recurrent or Metastatic Epidermal Growth Factor Receptor and/or erbB2 Expressing Adenoid Cystic Carcinoma and Non–Adenoid Cystic Carcinoma Malignant Tumors of the Salivary Glands. J. Clin. Oncol..

[93] Dillon P.M., Petroni G.R., Horton B.J., Moskaluk C.A., Fracasso P.M., Douvas M.G., Varhegyi N., Zaja-Milatovic S., Thomas C.Y. (2017). A Phase II Study of Dovitinib in Patients with Recurrent or Metastatic Adenoid Cystic Carcinoma. Clin. Cancer Res..

[94] Locati L.D., Galbiati D., Calareso G., Alfieri S., Singer S., Cavalieri S., Bergamini C., Bossi P., Orlandi E., Resteghini C. (2020). Patients with adenoid cystic carcinomas of the salivary glands treated with lenvatinib: Activity and quality of life. Cancer.

[95] Tchekmedyian V., Sherman E.J., Dunn L., Tran C., Baxi S., Katabi N., Antonescu C.R., Ostrovnaya I., Haque S.S., Pfister D.G. (2019). Phase II Study of Lenvatinib in Patients With Progressive, Recurrent or Metastatic Adenoid Cystic Carcinoma. J. Clin. Oncol..

[96] Pfister D.G., Spencer S., Adelstein D., Adkins D., Anzai Y., Brizel D.M., Bruce J.Y., Busse P.M., Caudell J.J., Cmelak A.J. (2020). Head and Neck Cancers, Version 2.2020, NCCN Clinical Practice Guidelines in Oncology. J. Natl. Compr. Canc. Netw..

[97] Ho A.L., Sherman E.J., Fury M.G., Baxi S.S., Haque S., Sima C.S., Antonescu C.R., Katabi N., Pfister D.G. (2014). Phase II study of axitinib in patients with progressive, recurrent/metastatic adenoid cystic carcinoma. J. Clin. Oncol..

[98] Ho A.L., Sherman E.J., Baxi S.S., Haque S., Ni A., Antonescu C.R., Katabi N., Morris L.G., Chan T.A.-T., Pfister D.G. (2016). Phase II study of regorafenib in progressive, recurrent/metastatic adenoid cystic carcinoma. J. Clin. Oncol..

[99] Thomson D.J., Silva P., Denton K., Bonington S., Mak S.K., Swindell R., Homer J., Sykes A.J., Lee L.W., Yap B.K. (2015). Phase II trial of sorafenib in advanced salivary adenoid cystic carcinoma of the head and neck. Head Neck.

[100] Guigay J., Fayette J., Even C., Cupissol D., Rolland F., Peyrade F., Laguerre B., Le Tourneau C., Zanetta S., Le Moal L.B. (2016). PACSA: Phase II study of pazopanib in patients with progressive recurrent or metastatic (R/M) salivary gland carcinoma (SGC). J. Clin. Oncol..

[101] Even C., Lassen U., Merchan J., Le Tourneau C., Soria J.C., Ferte C., Ricci F., Diener J.T., Yuen E., Smith C. (2020). Safety and clinical activity of the Notch inhibitor, crenigacestat (LY3039478), in an open-label phase I trial expansion cohort of advanced or metastatic adenoid cystic carcinoma. Investig. New Drugs.

[102] Ferrarotto R., Eckhardt G., Patnaik A., LoRusso P., Faoro L., Heymach J., Kapoun A., Xu L., Munster P. (2018). A phase I dose-escalation and dose-expansion study of brontictuzumab in subjects with selected solid tumors. Ann. Oncol..

[103] Miranda E.L., Stathis A., Hess D., Racca F., Quon D., Rodon J., Gadea O.S.S., Garcia J.M.P., Nuciforo P., Vivancos A. (2021). Phase 1 study of CB-103, a novel first-in-class inhibitor of the CSL-NICD gene transcription factor complex in human cancers. J. Clin. Oncol..

[104] Ayala Pharmaceuticals I. A Study of AL101 in Patients with Adenoid Cystic Carcinoma (ACC) Bearing Activating Notch Mutations (ACCURACY). https://clinicaltrials.gov/ct2/show/NCT03691207b.

[105] Kim D.-W., Oh D.-Y., Shin S.H., Kang J.H., Cho B.C., Chung J.-S., Kim H., Park K.U., Kwon J.H., Han J.-Y. (2014). A multicenter phase II study of everolimus in patients with progressive unresectable adenoid cystic carcinoma. BMC Cancer.

[106] Fayette J., Even C., Digue L., Geoffrois L., Rolland F., Cupissol D., Guigay J., Le Tourneau C., Dillies A.-F., Zanetta S. (2019). NISCAHN: A phase II, multicenter nonrandomized trial aiming at evaluating nivolumab (N) in two cohorts of patients (pts) with recurrent/metastatic (R/M) salivary gland carcinoma of the head and neck (SGCHN), on behalf of the Unicancer Head & Neck Group. J. Clin. Oncol..

[107] Cohen R.B., Delord J.P., Doi T., Piha-Paul S.A., Liu S.V., Gilbert J., Algazi A.P., Damian S., Hong R.L., Le Tourneau C. (2018). Pembrolizumab for the treatment of advanced salivary gland carcinoma: Findings of the phase 1b KEYNOTE-028 study. Am. J. Clin. Oncol..

[108] Mahmood U., Bang A., Chen Y.-H., Mak R.H., Lorch J.H., Hanna G.J., Nishino M., Manuszak C., Thrash E.M., Sev- ergnini M. (2020). A Randomized Phase 2 Study of Pembrolizumab with or Without Radiation in Patients with Recurrent or Metastatic Adenoid Cystic Carcinoma. Int. J. Radiat. Oncol..

[109] Wang Y., Cai S., Xue Q., Mu J., Gao Y., Tan F., Mao Y., Wang D., Zhao J., Gao S. (2020). Treatment outcomes of patients with tracheobronchial mucoepidermoid carcinoma compared with those with adenoid cystic carcinoma. Eur. J. Surg. Oncol. EJSO.

[110] Chen Z., Ni W., Li J.-L., Lin S., Zhou X., Sun Y., Li J.W., Leon M.E., Hurtado M.D., Zolotukhin S. (2021). The CRTC1-MAML2 fusion is the major oncogenic driver in mucoepidermoid carcinoma. J. Clin. Investig..

[111] Tonon G., Modi S., Wu L., Kubo A., Coxon A.B., Komiya T., O’Neil K., Stover K., El-Naggar A., Griffin J.D. (2003). t(11;19)(q21;p13) translocation in mucoepidermoid carcinoma creates a novel fusion product that disrupts a Notch signaling pathway. Nat. Genet..

[112] Chen Z., Chen J., Gu Y., Hu C., Li J.-L., Lin S., Shen H., Cao C., Gao R., Ha P.K. (2014). Aberrantly activated AREG–EGFR signaling is required for the growth and survival of CRTC1–MAML2 fusion-positive mucoepidermoid carcinoma cells. Oncogene.

[113] Canettieri G., Coni S., Della Guardia M., Nocerino V., Antonucci L., Di Magno L., Screaton R., Screpanti I., Giannini G., Gulino A. (2009). The coactivator CRTC1 promotes cell proliferation and transformation via AP-1. Proc. Natl. Acad. Sci. USA.

[114] Amelio A.L., Fallahi M., Schaub F.X., Zhang M., Lawani M.B., Alperstein A.S., Southern M.R., Young B.M., Wu L., Zajac-Kaye M. (2014). CRTC1/MAML2 gain-of-function interactions with MYC create a gene signature predictive of cancers with CREB-MYC involvement. Proc. Natl. Acad. Sci. USA.

[115] Wu L., Liu J., Gao P., Nakamura M., Cao Y., Shen H., Griffin J.D. (2005). Transforming activity of MECT1-MAML2 fusion oncoprotein is mediated by constitutive CREB activation. EMBO J..

[116] Ni W., Chen Z., Zhou X., Yang R., Yu M., Lu J., Kaye F.J., Wu L. (2021). Targeting Notch and EGFR signaling in human mucoepidermoid carcinoma. Signal Transduct. Target. Ther..

[117] Reya T., Morrison S.J., Clarke M.F., Weissman I.L. (2001). Stem cells, cancer, and cancer stem cells. Nature.

[118] Wang K., McDermott J.D., Schrock A.B., Elvin J.A., Gay L., Karam S.D., Raben D., Somerset H., Ali S.M., Ross J.S. (2017). Comprehensive genomic profiling of salivary mucoepidermoid carcinomas reveals frequent BAP1, PIK3CA, and other actionable genomic alterations. Ann. Oncol..

[119] Locati L., Perrone F., Cortelazzi B., Bergamini C., Bossi P., Civelli E.M., Morosi C., Vullo S.L., Imbimbo M., Quattrone P. (2016). A phase II study of sorafenib in recurrent and/or metastatic salivary gland carcinomas: Translational analyses and clinical impact. Eur. J. Cancer.

[120] Kim Y., Lee S.J., Lee J.Y., Lee S.-H., Sun J.-M., Park K., An H.J., Cho J.Y., Kang E.J., Lee H.-Y. (2017). Clinical trial of nintedanib in patients with recurrent or metastatic salivary gland cancer of the head and neck: A multicenter phase 2 study (Korean Cancer Study Group HN14-01). Cancer.

[121] Gaissert H.A., Burns J. (2010). The Compromised Airway: Tumors, Strictures, and Tracheomalacia. Surg. Clin. North. Am..

[122] Urdaneta A.I., Yu J.B., Wilson L.D. (2011). Population Based Cancer Registry Analysis of Primary Tracheal Carcinoma. Am. J. Clin. Oncol..

[123] Xie L., Fan M., Sheets N.C., Chen R.C., Jiang G.-L., Marks L.B. (2012). The Use of Radiation Therapy Appears to Improve Outcome in Patients with Malignant Primary Tracheal Tumors: A SEER-Based Analysis. Int. J. Radiat. Oncol..

[124] Reck M., Rodríguez-Abreu D., Robinson A.G., Hui R., Csőszi T., Fülöp A., Gottfried M., Peled N., Tafreshi A., Cuffe S. (2016). Pembrolizumab versus Chemotherapy for PD-L1–Positive Non–Small-Cell Lung Cancer. N. Engl. J. Med..

[125] Lopes G., Wu Y.-L., Kudaba I., Kowalski D., Cho B.C., Castro G., Srimuninnimit V., Bondarenko I., Kubota K., Lubiniecki G.M. (2018). Pembrolizumab (pembro) versus platinum-based chemotherapy (chemo) as first-line therapy for advanced/metastatic NSCLC with a PD-L1 tumor proportion score (TPS) ≥ 1%: Open-label, phase 3 KEYNOTE-042 study. J. Clin. Oncol..

[126] Paz-Ares L., Luft A., Vicente D., Tafreshi A., Gümüş M., Mazières J., Hermes B., Çay Şenler F., Csőszi T., Fülöp A. (2018). KEYNOTE-407 Investigators. Pembrolizumab plus Chemotherapy for Squamous Non-Small-Cell Lung Cancer. N. Engl. J. Med..

[127] Gandara D.R., Hammerman P.S., Sos M.L., Lara P.N., Hirsch F.R. (2015). Squamous Cell Lung Cancer: From Tumor Genomics to Cancer Therapeutics. Clin. Cancer Res..

[128] Paik P.K., Pillai R.N., Lathan C.S., Velasco S.A., Papadimitrakopoulou V. (2019). New Treatment Options in Advanced Squamous Cell Lung Cancer. Am. Soc. Clin. Oncol. Educ. Book.

[129] Bahleda R., Dienstmann R., Adamo B., Gazzah A., Infante J.R., Zhong B., Platero S.J., Smit H., Perera T., Stuyckens K. (2014). Phase 1 study of JNJ-42756493, a pan-fibroblast growth factor receptor (FGFR) inhibitor, in patients with advanced solid tumors. J. Clin. Oncol..

[130] Nogova L., Sequist L.V., Cassier P.A., Hidalgo M., Delord J.-P., Schuler M.H., Lim W.-T., Camidge D.R., Buettner R., Heukamp L.C. (2014). Targeting *FGFR1*-amplified lung squamous cell carcinoma with the selective pan-FGFR inhibitor BGJ398. J. Clin. Oncol..

[131] Spoerke J.M., O’Brien C., Huw L., Koeppen H., Fridlyand J., Brachmann R.K., Haverty P.M., Pandita A., Mohan S., Sampath D. (2012). Phosphoinositide 3-Kinase (PI3K) Pathway Alterations Are Associated with Histologic Subtypes and Are Predictive of Sensitivity to PI3K Inhibitors in Lung Cancer Preclinical Models. Clin. Cancer Res..

[132] Vansteenkiste J.F., Canon J.L., De Braud F., Grossi F., De Pas T., Gray J.E., Su W.-C., Felip E., Yoshioka H., Gridelli C. (2015). Safety and efficacy of buparlisib (BKM120) in patients with PI3K pathway-activated non-small cell lung cancer: Results from the phase II BASALT-1 study. J. Thorac. Oncol..

[133] Bendell J.C., Varghese A.M., Hyman D.M., Bauer T.M., Pant S., Callies S., Lin J., Martinez R., Wickremsinhe E.R., Fink A. (2018). A First-in-Human Phase 1 Study of LY3023414, an Oral PI3K/mTOR Dual Inhibitor, in Patients with Advanced Cancer. Clin. Cancer Res..

[134] Wade J.L., Langer C.J., Redman M., Aggarwal C., Bradley J.D., Crawford J., Miao J., Griffin K., Herbst R.S., Kelly K. (2017). A phase II study of GDC-0032 (taselisib) for previously treated PI3K positive patients with stage IV squamous cell lung cancer (SqNSCLC): LUNG-MAP sub-study SWOG S1400B. J. Clin. Oncol..

[135] Li J., Tan F., Wang Y., Xue Q., Gao Y., Mu J., Mao Y., Zhao J., Wang D., Feng X. (2022). Clinical characteristics, surgical treatments, prognosis, and prognostic factors of primary tracheal cancer patients: 20-year data of the National Cancer Center, China. Transl. Lung Cancer Res..

[136] Genden E.M., Laitman B.M. (2023). Human Tracheal Transplantation. Transplantation.

[137] Zheng Z., Du Z., Fang Z., Shi Y., Chen X., Jin M., Liu K. (2023). Survival benefit of radiotherapy and nomogram for patients with primary tracheal malignant tumors: A propensity score-matched SEER database analysis. J. Cancer Res. Clin. Oncol..

